# Adaptive Behavior as an Alternative Outcome to Intelligence Quotient in Studies of Children at Risk: A Study of Preschool-Aged Children in Flint, MI, USA

**DOI:** 10.3389/fpsyg.2021.692330

**Published:** 2021-08-11

**Authors:** Shuting Zheng, Kaja LeWinn, Tiffany Ceja, Mona Hanna-Attisha, Lauren O'Connell, Somer Bishop

**Affiliations:** ^1^Department of Psychiatry and Behavioral Sciences, Weill Institute for Neurosciences, University of California, San Francisco, San Francisco, CA, United States; ^2^Division of Public Health, Pediatric Public Health Initiative, Michigan State University, Flint, MI, United States; ^3^Department of Pediatrics and Human Development, Michigan State University, East Lansing, MI, United States

**Keywords:** adaptive behavior, IQ, nurturance, maternal education, modifiable predictors

## Abstract

Intelligence quotient (IQ) is commonly measured in child development studies, while adaptive behavior is less frequently considered. Given its associations with functional outcomes in children with neurodevelopmental disabilities, adaptive behavior may be a useful outcome in general population samples, as well. This study aimed to compare social and environmental correlates of adaptive behavior vs. IQ in a sample of preschoolers exposed to the Flint water crisis (*N* = 184). Mother–child dyads were recruited from the community and administered a comprehensive battery to obtain information about child neurodevelopmental functioning, including direct assessment of IQ *via* the Wechsler Preschool and Primary Scale of Intelligence and assessment of parent-reported adaptive functioning *via* the Vineland Adaptive Behavior Scales. Multiple social environmental factors were explored as potential correlates of child outcomes (i.e., IQ and adaptive behavior), and robust correlates were identified using a data-driven approach [i.e., least absolute shrinkage and selection operator (LASSO) regression]. We then examined associations between the LASSO-selected predictors and IQ and adaptive behavior while controlling for child age, child sex, and maternal age. Children in this sample showed relative strength in adaptive behaviors, with scores in the adequate range, while average IQs fell in the low-average range. Adaptive behavior was significantly associated with maternal nurturance practices, while IQ was associated with the maternal education level. Implications for the use of adaptive behavior as an outcome measure in studies of children at an increased risk for neurodevelopmental problems are discussed.

## Introduction

Adaptive behavior is defined as the conceptual, social, and practical skills that are needed to function within his/her environment of an individual in everyday life (Schalock et al., 2021). Historically, adaptive behavior has been a central point of discussion for nosology and outcomes in individuals with intellectual and developmental disabilities (Luckasson et al., 2002; National Research Council (US) Committee on Disability Determination for Mental Retardation et al., 2002; Alexander and Reynolds, 2020). In fact, while evidence of a low intelligence quotient (IQ) is still required for a diagnosis of intellectual disability (ID; previously called mental retardation), DSM-5 currently stipulates that the level of ID (i.e., mild, moderate, severe, and profound) should be based on adaptive functioning rather than IQ (American Psychiatry Association, 2013). This reflects the understanding that although cognitive and adaptive functioning is correlated, the capacity to acquire a given skill may be different than the likelihood of actually executing that skill in everyday life (Sparrow and Cicchetti, 1985; Keith et al., 1987; Oakland and Harrison, 2008; Alexander and Reynolds, 2020). For example, multiple studies of autism spectrum disorder (ASD) show that adaptive behavior can be significantly impaired even among individuals with high IQ (Klin et al., 2007; Duncan and Bishop, 2013; Kraper et al., 2017; Meyer et al., 2018). Furthermore, among adults with neurodevelopmental disabilities, especially those with ASD without ID, it is adaptive behavior (and not IQ) that is most associated with functional outcomes (Farley et al., 2009; Woolf et al., 2010; Taylor and Mailick, 2014; Taylor et al., 2015; Bishop-Fitzpatrick et al., 2016). Information about the relationship between IQ and adaptive behavior in typically developing populations is more limited and comes mainly from validation studies of adaptive behavior measures showing that, as intended, IQ and adaptive behavior are only moderately correlated (Sparrow et al., 2005, 2016; Harrison and Oakland, 2015).

In young children, adaptive behavior measures consider a wide range of developmentally relevant constructs known to affect early childhood outcomes, such as executive functioning, behavioral inhibition, social-emotional skills, and pre-academic skills (Luckasson et al., 2002; Sparrow et al., 2005; Oakland and Harrison, 2008). Thus, adaptive behavior may serve as a proximal indication of how an individual functions within developmentally relevant contexts and provide additional information important for conceptualizing the profile of risk of an individual child and resilience in their specific environment (Test et al., 2009; Bal et al., 2015; Dell'Armo and Tassé, 2019). In addition, adaptive behavior has been shown to be amenable to treatment in children with developmental disabilities (Matson et al., 2012; Bal et al., 2015; Duncan et al., 2018), making it a particularly appealing endpoint for targeted interventions.

Intelligence quotient is commonly included as both a predictor and an outcome in epidemiological research on child development (Halle et al., 2009; Calvin et al., 2017). Studies have shown that IQ is predictive of many important developmental outcomes, including language ability and academic performance (Neisser et al., 1996; Mayes et al., 2009; Duckworth et al., 2011). However, rather than providing a “pure” measure of ability, IQ scores may reflect a multitude of factors beyond the innate cognitive capacity of an individual (Croizet and Dutrévis, 2004; Fagan and Holland, 2007; Kendler et al., 2015; Ritchie and Tucker-Drob, 2018). For example, IQ has consistently been shown to be related to socioeconomic status (SES) variables, especially maternal education and household income levels (Duncan and Brooks-Gunn, 2000; National Institute of Child Health Human Development Early Child Care Research Network, 2005; Nelson et al., 2007; Tucker-Drob et al., 2013; Kendler et al., 2015; LeWinn et al., 2020). Moreover, as a measured construct, IQ has a number of well-known limitations that have sparked historical debate and controversy. Specifically, early studies introduced serious questions about why racially and culturally diverse groups scored lower in the knowledge of learned information on IQ measures and raised concerns about cross-cultural validity of IQ tests given cultural differences in conceptualizations of intelligence (Jensen, 1980; Helms, 1992; Rushton and Jensen, 2005; Sternberg et al., 2005; Fagan and Holland, 2007). In contrast, although not independent of cultural and contextual expectations about development, adaptive behavior measures may be less susceptible to systematic biases related to SES or race/ethnicity because of the focus on everyday functioning within his/her own environment of an individual (Reschly et al., 2002a). However, given the limited research on adaptive behavior in general population samples, much less is known about adaptive behavior correlates, or how these correlates differ from those of IQ. This information is essential to inform choices of meaningful outcomes in children from diverse backgrounds.

As mentioned above, the majority of research on adaptive behavior has been conducted within clinical populations of children and adults with intellectual and developmental disabilities (Ditterline et al., 2008). This study has focused mainly on individual-level predictors of adaptive behavior and has identified IQ, language, and executive functioning as significant (Kanne et al., 2011; Ware et al., 2012; Bal et al., 2015; Pugliese et al., 2015; Gardiner and Iarocci, 2018; Bertollo et al., 2020). Relatively few studies have examined how the aspects of the social environment are related to adaptive behavior skills (Glaser et al., 2003). However, a handful of studies have shown that maternal responsivity and growth facilitating behaviors (e.g., basic care and learning activities) promote adaptive skills in very young children (Altman and Mills, 1990) and children with developmental delays (Fenning and Baker, 2012; Warren et al., 2017). These findings suggest that understanding the influences of modifiable social environmental factors, including factors related to parent–child interactions, could have important implications for interventions designed to improve adaptive behavior.

This study was conducted to examine the correlates of IQ and adaptive behavior in a group of non-clinically referred preschoolers from Flint, MI, USA. All children in this study were postnatally exposed to the Flint water crisis, which began in April 2014 and imposed unprecedented trauma on the Flint community with lead exposure and increased stress related to water use and beyond (Hanna-Attisha et al., 2015; Ruckart et al., 2019). Thus, these children experienced myriad socioeconomic and environmental exposures (e.g., racism, poverty, and lead) during the first few years of life. Comprehensive assessments were conducted to assess the neurodevelopmental functioning of children across multiple domains, with previous analyses by our group showing highly variable developmental profiles within the sample at the age of 4 years (Zheng et al., 2021). Building on this work, we employed a data-driven approach to explore associations between a broad range of social and environmental predictors and two main child outcomes of interests, namely, IQ and adaptive behavior. This study was motivated by an interest in identifying potentially modifiable correlates of IQ and adaptive behavior, with a particular goal of understanding the utility of measuring adaptive behavior as an alternative outcome in high-risk samples like those of ours.

## Method

### Participants

Mother–child dyads were invited to participate if the child was born between March 1, 2012 and April 24, 2014 (before the water source change) and if the child resided in the City of Flint and received water from the Flint water distribution system between April 25, 2014 and October 15, 2015. Children who fit these inclusion criteria would have been exposed to the water within the first 2 years of life and were old enough to complete direct assessments of developmental domains of interest at the in-person visit.

Families who expressed interest in participation were screened for eligibility. Children were excluded if they were wards of the state, if their birth weight was <1,500 g, if their gestational age was <32 weeks, or if they had a known genetic syndrome. Mother–child dyads were only included if the caregiver of the eligible child was their biological mother, spoke English, and reside with and consistently care for the child. To ensure that they could validly complete the tests included in the direct assessment battery, children were also excluded if they were currently non-verbal or had significant hearing or visual impairments. A total of 390 mother–child dyads participated in screening, of whom 284 dyads were determined to be eligible and 272 agreed to enroll. A total of 184 families attended an in-person assessment, of whom 157 completed the Vineland Adaptive Behavior Scale and 174 completed the IQ test. The characteristics of the full sample are shown in Table 1.

**Table 1:** Demographic characteristics of the current sample (*N* = 184).

**Variables**	**Categories**	***N***	**%**
Child gender	Male	100	54.4%
	Female	84	45.6%
Child race	White	28	15.2%
	Black or African American	125	67.9%
	Other	3	1.6%
	Multi-race	23	12.6%
	Missing	5	2.7%
Child ethnicity	Non-Hispanic	165	89.7%
	Hispanic	19	10.3%
Maternal education level	Less than high school	22	12.0%
	High school or GED	67	36.4%
	Vocational or technical school	5	2.7%
	Some college or associate degree	69	37.5%
	College graduate or bachelor's degree	12	6.5%
	Graduate degree	4	2.2%
	Missing	5	2.7%
Maternal employment	Employed and working	95	51.6%
	Not employed	83	45.1%
	Missing	6	3.3%
Household income level	< $10,000	62	33.7%
	$10,000–$15,000	34	18.5%
	$15,001–$25,000	21	11.4%
	$25,001–$35,000	16	8.7%
	$35,001–$45,000	9	4.9%
	More than $45,000^a^	17	9.2%
	Don't know	16	8.70%
	Missing	9	4.89%
Number of children in the household	1	36	19.6%
	2	47	25.5%
	3	50	27.2%
	≥4	41	22.3%
	Missing	10	5.4%
	***N***	**M (SD)**	**Range**
Child age	184	5.48(0.38)	[4.05, 6.22]
Maternal age	184	31.46(6.32)	[21.25, 52.95]

a *Given the small numbers in each of the below categories, we collapsed multiple categories into one: $45,001–$55,000, $55,001–$65,000, $65,001–$75,000, $75,001–$100,000, $100,001–$150,000, $150,001–$200,000, $200,001, or more*.

### Procedure

Once eligibility was established, mothers completed online and in-person surveys, and children completed direct in-person assessments. For mothers who expressed any difficulty with reading or seemed to struggle to understand the questions, trained research staff were available to read the questions and record their responses (on the phone or in person) to ensure the validity of their report and minimize barriers to participating. All research assistants involved in the assessments received training and supervision in the administration of study measures from a licensed clinical psychologist. The institutional review board at the institutions of authors reviewed and approved the study protocol, and the Michigan Department of Health and Human Services and Hurley Medical Center approved the affiliated recruitment protocol. Informed consent was obtained from mothers and verbal assent was obtained from children before the beginning of participation.

### Measures

The two main child outcomes of interest for this study were measured with widely used standardized measures. IQ was measured using the *Wechsler Preschool and Primary Scale of Intelligence–Fourth Edition (WPPSI–IV)*, a commonly used intelligence test designed for children aged 2 years, between 6 months and 7 years, and 7 months (Wechsler, 2012). The current analysis used the norm-referenced standard scores corresponding to full-scale IQ (FSIQ) with a mean of 100 and an standard deviation (SD) of 15.

The *Vineland Adaptive Behavior Scales (Sparrow et al.*, *2005*, *2016**; Vineland 3; Vineland-II)* was used to measure the adaptive behavior skills. Adaptive behavior measures, such as the Vineland, involve clinical interviews or checklists completed by informants who have regular opportunities to directly observe adaptive behaviors performed within the everyday environment of an individual (Reschly et al., 2002b; Tassé et al., 2012; Harrison and Oakland, 2015). Given that this study focused on preschool-aged children who typically spend the majority of time with primary caregivers, mothers served as the informant about the adaptive behavior skills of children. Because of protocol changes that occurred mid-study, some mothers completed the Vineland-II comprehensive interview form (*N* = 40) and others completed the Vineland-3 online parent-report form (*N* = 117). Both versions yield an adaptive behavior composite (ABC) score representing the overall level of adaptive functioning, which was used in the current analysis.

Social environmental exposures of interest were collected from parents *via* interviews and questionnaires. These variables were selected given demonstrated associations with child neurodevelopmental outcomes and because they could be considered modifiable by programs, practices, or policies (the detailed descriptions of each measure and example citations showing their associations with child outcomes are shown in Table 2).

**Table 2:** Description of exposure measures included.

**Measure**	**Summary**	**Variable entered in LASSO**	Example citations^*^
Center for Epidemiological Studies-Depression Scale (CES-D) Radloff (1977)	The CES-D consists of 20 self-report items assessing for depression. Each item is rated on a four-point scale from “Rarely or none of the time (<1 day)” to “Most or all of the time (5–7 days)” over the past week. Higher scores on the CES-D indicate higher levels of depressive symptoms, with a cut-off of 16 denoting clinically significant depressive symptoms Lewinsohn et al. (1997).	Standardized CES-D summary score	Murray et al., 1996; Barker et al., 2011; Huang et al., 2014; Bush et al., 2020
Perceived Stress Scale (PSS) Cohen et al. (1983)	The PSS includes ten items on a five-point scale form “Never” to “Very often,” measuring perceived stress during the last month. Total scores on PSS were calculated for analysis, with higher scores indicating higher stress.	Standardized PSS total score	Weinraub and Wolf, 1983; Keim et al., 2011; Huang et al., 2014; LeWinn et al., 2020
The CAGE Adapted to Include Drugs (CAGE-AID) Brown and Rounds (1995)	The CAGE–AID is used to screen the respondent for problems associated with drug abuse. The assessment is self–completed and consists of four yes/no questions. The participant is asked about cutting down (or feeling they should cut back on drug use), annoyance (whether others have been annoyed with the participant's drug use), feeling guilty (about drug use), and the use of “eye–openers” (feeling the need to use drugs upon waking in the morning).	Standardized CAGE-AID total score	Blanchard et al., 2005
Life Orientation Test-Revised (LOT-R) Scheier et al. (1994)	The Life Orientation test is used to assess the dispositional optimism (the general expectancy of positive outcomes) and psychological resilience of the respondent. It is a self–reported test containing 10 items. Three of these items are positively worded, three are negatively worded, and the remaining four are fillers. Each item is rated on a four–point scale (0 = strongly disagree, 1 = disagree, 2 = neutral, 3 = agree, and 4 = strongly agree). The negative items are reverse scored, and the fillers are left unscored.	Standardized LOT total score	Baker et al., 2005
Social Support Questionnaire (SSQ) Sarason et al. (1983)	This questionnaire is used to measure the respondent's perceived level of social support and to measure their satisfaction with this support. For each item, the respondent must list all of those who fit the description of the question, describe their relation to the person listed, then rate how satisfied they are with these relationships on a six–point scale (1 = very dissatisfied, 6 = very satisfied). The version adapted by the study contains 6 items, asking the respondent who in their lives they find dependable, helps them relax, accepts them fully, cares about them, helps them feel better, and consoles them.	Standardized SSQ support level score and satisfaction score	Weinraub and Wolf, 1983; Burchinal et al., 1996; Huang et al., 2014
HARK 4 item Sohal et al. (2007)	The HARK questions are used to identify victims of intimate partner violence (IPV). HARK is an acronym for Humiliation, Afraid, Rape, Kick, and each of the four items in the test pertains to one of these categories (humiliation, intimidation, sexual assualt, and physical abuse). The questions are in yes/no format and one point is given for every “yes” answer. Studies show the test to accurately identify women experiencing IPV.	Standardized HARK sum total score	Harding et al., 2013; Vu et al., 2016
Child Rearing Practices Report (CRPR): nurturance and conflict subscale Rickel and Biasatti (1982)	The CPRP evaluates the goals, values, and attitudes of parents in regards to raising a child. The Nurturance subscale of the test adapted by the study specifically evaluates the respondent's level of affection, attention, and nurturance that they provide to their child. This subscale contains 18 items. The conflict subscale is used to assess the level of conflict between the respondent and their child. This subscale contains 3 items. Both subscales are parent–completed and each question is rated on a six–point scale (1 = Not at all descriptive of me, 6 = highly descriptive of me). The CPRP is shown to reliably predict children's future adaptation Gerhardt et al. (2003)	Standardized CRPR Nurturance summary total score Principal Component Score of the three conflict items	Farah et al., 2008; Rochelle and Cheng, 2016; Bush et al., 2020; LeWinn et al., 2020
Knowledge of Effective Parenting Scale (KEPS) Winter et al. (2012)	The KEPS is used to measure the respondent's knowledge of effective parenting strategies for parents of children aged 2–10. The test adapted by the study contains 17 items, all of which are multiple–choice format. These items address four main areas of parental knowledge: promotion of development, principles of effective parenting, use of assertive discipline, and causes of behavior problems Winter et al. (2012). One point is given to the respondent for each question answered correctly. Previous studies have shown that the test has good content validity, satisfactory test–retest reliability, and internal consistency Winter et al. (2012).	Standardized KEPS score	Winter et al., 2012; Rochelle and Cheng, 2016
Network of Relationship Inventory (NRI)—Criticism Scale (revised parent version) Furman and Buhrmester (1985)	The adapted NRI is used to evaluate the frequency of criticism and harsh feedback that the respondent gives to their child. The survey is parent–completed and contains three items measured on a five–point scale (Little or none, Somewhat, Very much, Extremely much, The most).	Standardized NRI average score across items	Harris and Howard, 1984; Jacquez et al., 2004; Wolford et al., 2019
StimQ-Parent (StimQ-P) read scale and parent verbal responsivity scale Mendelson et al. (2016)	This test evaluates cognitive stimulation in the home environment for children aged 36–60 months. The questionnaire is parent–completed and consists of mostly “yes or no” questions. The StimQ—P2 contains four subscales: Availability of Learning Materials, Reading (“reflecting access to books, frequency of shared reading, variety of books read, and interactivity/quality of reading”), Parental Involvement in Developmental Advance, and Parental Verbal Responsivity. The version adapted for the study solely includes questions regarding book reading quantity, book reading quality, and parental responsivity (during everyday routines, during play, and during activities that promote regulation).	Standardized StimQ-P total score	Rodriguez and Tamis-LeMonda, 2011; Baker, 2013; Malhi et al., 2018; LeWinn et al., 2020
National Survey of Children's Health ACE questions Bethell et al. (2017)	This 8-item questionnaire assesses the health and well–being of children aged 0–17 based on the amount of reported Adverse Childhood Experiences (ACEs). These questions assess whether the child has witnessed and/or experienced parental divorce, death of a parent, parental incarceration, domestic abuse, neighborhood violence, household mental illness, household substance abuse, and racial discrimination. Studies consistently show that an increasing number of ACEs has a strong relationship with poorer health outcomes. However, the ACE assessment is not recommended to be used as a diagnostic tool, but to open dialogue and to indicate a need for further evaluation Bethell et al. (2017).	Standardized total number of ACEs reported	Crouch et al., 2019
Demographics survey	The purpose of this questionnaire is to gather information about the background and living arrangements of the respondent and their child. Specifically, the child's and mother's age, sex, and race. The survey then addresses information specifically regarding the parent's background, such as educational history, employment status, and marriage status, household income levels, and number of children in the household.	Maternal education level Maternal relationship status Maternal employment statusHousehold income level Number of children in the household	Duncan et al., 1994; Noble et al., 2005; Bush et al., 2020; LeWinn et al., 2020

* *Previous studies showing associations of the measured construct/exposure with child outcomes*.

*Maternal characteristics* included depressive symptoms measured by the Center for Epidemiological Studies Depression Scale (Radloff, 1977), stress measured by the Perceived Stress Scale (Cohen et al., 1983), potential problems with substance use was measured by the CAGE Adapted to Include Drugs screener (Brown and Rounds, 1995), and dispositional optimism measured by the Life Orientation Test-Revised (Scheier et al., 1994). Maternal perceived social support was measured by the Social Support Questionnaire (Sarason et al., 1983), and domestic violence was measured using the 4-item HARK (Sohal et al., 2007).

*Parenting measures* included the Child Rearing Practices Report (CRPR) nurturance and conflict subscales (Rickel and Biasatti, 1982), the Knowledge of Effective Parenting Scale (Winter et al., 2012), the Network of Relationship Inventory-Criticism Scale (revised parent version; Furman and Buhrmester, 1985), and the stimulation questionnaire (StimQ-Parent; Mendelson et al., 2016; Read Scale and Parent Verbal Responsivity Scale).

*Early childhood experiences* were measured using the National Survey of Children's Health adverse childhood experiences questions (Bethell et al., 2017).

*Sociodemographic characteristics*, including highest maternal education, maternal relationship status, maternal employment status, and annual household income and household size, were collected through surveys completed by mothers. Specifically, maternal relationship status was coded as single vs. partnered/in a relationship, and maternal employment status was coded as working (including full-time and part-time) vs. not working (including unemployed and retired). Since most of the mothers were either high school graduates or had completed some college education (Table 1), we coded maternal education level as high school graduate and below vs. some college and above. Regarding household income, we adopted the Organization for Economic Co-operation and Development (OECD)-modified equivalence scale to adjust the income level based on the household sizes: first, the household size was determined by assigning a value of 1 to the household head, 0.5 to each additional adult member, and 0.3 to each child (Organisation for Economic Co-operation Development, 2021); then, we took the medians of the income categories (e.g., for category $15,001–$25,000, median $17,500 was used, $5,000 was used for the “ < $10,000” category, and $200,000 used for “$200,000 and more”) to be divided by the household sizes to generate the OECD-modified income level; finally, the number of children in the household was included as a categorical variable with four classes, namely, 1, 2, 3, and ≥4.

*A priori identified confounders* included child age, child sex at birth, and maternal age.

### Analysis Plan

Descriptive statistics (i.e., mean and SD) for the primary child outcomes, predictors, and confounding variables of interest were generated (see Table 1 for demographic variables and Supplementary Table 1 for descriptive statistics on maternal characteristics and parenting measures). For the regression analysis, summary scores of the measures were used. When a summary score was not available (i.e., the child-rearing practices–conflict subscale), the principal component analysis was conducted to generate a single score to be included in the regression models.

#### Predictor Selection

We applied the least absolute shrinkage and selection operator (LASSO) method to select predictors from the full list of target exposures (*N* = 18) to be included in the regression models predicting adaptive behavior skills and IQ levels (see Table 2 for variables entered in LASSO regression). The LASSO method offers the advantage of selecting stable predictors and excluding factors with nominal effects and collinear covariates. All continuous variables were standardized with a mean of 0 and an SD of 1 to be on the same scale of influence on the penalty term in the LASSO model. In this analysis, we applied 5-fold external cross-validation for determining the LASSO model with model selection based on the fit indicator of predicted residual sum of square (PRESS) for *k*-fold external cross-validation. Furthermore, due to concerns of overfitting with a relatively small sample and to achieve higher confidence for predictor selection, we conducted LASSO modeling with different partitioning with a random selection of training and testing samples from the full sample to examine the resulting model as follows: (1) LASSO models run with the full sample used for training, (2) LASSO models run in 90% of the sample for training and 10% for testing, (3) LASSO models run in 80% of the sample for training and 20% for testing, and (4) LASSO models run in 70% of the sample for training and 30% for testing. LASSO selection results from each model were presented and compared with fit indices of the Akaike Information Criterion, PRESS, and average squared error of the training and testing samples. Separate LASSO regressions were run to select the sets of reliable predictors of adaptive behavior and IQ.

#### Post-LASSO Regression Models

In the post-LASSO analysis, the multiple linear regressions with standardized scores were fitted to estimate the magnitude of coefficients associated with predictors of adaptive behaviors and IQ separately while controlling for *a priori* identified confounders. Predictor(s) that were consistently selected across LASSO models for either adaptive behaviors or IQ were entered into respective linear models first, and then the other predictors selected inconsistently by LASSO models were entered at a later step. Coefficients of LASSO-selected predictors were estimated adjusting for confounders identified *a priori* (i.e., child age, child sex at birth, and maternal age). For adaptive behavior and IQ models, we reported the standardized parameter estimates (β) with 95% confidence limits (CL), effect sizes (partial η^2^), and *p*-values for all the predictor variables and possible confounders at each step, and *R*^2^ and *R*^2^ changes of the models.

## Results

Flint preschoolers in the current sample showed a wide range of adaptive behavior and IQ scores, with the mean level of adaptive behaviors falling within the adequate range based on the norm-referenced ABC score (*M* = 94.67, SD = 15.73, IQR = 19, range: 48–140) and the mean FSIQ falling in the low average range (*M* = 88.07, SD = 12.48, IQR = 20, range = 62–120). Vineland ABC scores and WPPSI FSIQ scores showed only a small correlation of 0.27. Measures of social-environmental characteristics (e.g., maternal mental health and parenting) showed a wide range of scores, spanning the full range for most of the measures. The descriptive statistics and correlation matrix are shown in (Supplementary Tables 1, 2).

### Predictors of IQ

The LASSO regression consistently selected the maternal education level as a predictor of child IQ across all models (Table 3). The other predictor identified by two out of four models was OECD-adjusted household income. When the maternal education level, together with *a priori* predictors (i.e., child sex, child age, and maternal age), were included in the same model, the maternal education level (β = 0.48, 95% CL: 0.19–0.77) and child sex (β = 0.47, 95% CL: 0.18–0.76) were significant (Table 4). Maternal education and child sex remained the significant predictors of IQ when OECD-adjusted income was added to this model (Δ*R*^2^ = 0.019; Table 4). Children with mothers who had received more than high school education showed IQ scores with an SD of 0.40 higher than those with mothers with lower educational levels, and girls received IQ scores with an SD of 0.45 higher than boys. The estimated coefficient of OECD-adjusted income level was not significant.

**Table 3:** Results from LASSO models with adaptive behavior and IQ as outcomes with different sample partitioning.

		**LASSO-selected predictors**	**AIC**	**Press**	**ASE for training sample**	**ASE for testing sample**
**IQ** LASSO results	Full sample training	**Maternal education**	78.765	0.943	–	–
	90% for training vs. 10% for testing	**Maternal education**, Income per capita	77.074	0.987	1.001	0.279
	80% for training vs. 20% for testing	**Maternal education**	68.545	0.944	1.026	0.882
	70% for training vs. 30% for testing	**Maternal education**, Income per capita	57.221	1.062	0.915	1.089
**Adaptive behavior** LASSO results	Full sample training	**CRPR nurturance, social support satisfaction**	72.164	0.874	–	–
	90% for training vs. 10% for testing	**CRPR nurturance**, **social support satisfaction**	69.337	1.002	0.884	0.695
	80% for training vs. 20% for testing	**CRPR nurturance**, **social support satisfaction**, Stimulating environment	54.330	0.807	0.752	1.062
	70% for training vs. 30% for testing	Intercept only	56.966	1.029	0.981	1.003

*AIC, Akaike information criterion; PRESS, predicted residual sum of square; ASE, average squared error*.

**Table 4:** Results from adjusted model of LASSO-selected predictors for IQ with standardized scores.

			**Model 1 (** ***N*** **=** **174)**			**Model 2 (** ***N*** **=** **142)**		
			**R-Square: 0.122** ***p*** **=** **0.0002**			**R-Square: 0.141** ***p<0.0001***		
			**β** **(95% CI)**	***p***	**Partial** **η^2^**	**β** **(95% CI)**	***p***	**Partial** **η^2^**
LASSO selected	**Maternal education**	**≤High school**	**[Reference]**	**–**	**–**	**[Reference]**	**–**	**–**
		**>High school**	**0.48 (0.19, 0.77)**	**0.001**	**0.06**	**0.40 (0.07, 0.72)**	**0.017**	**0.04**
		Household income				0.15 (−0.00, 0.31)	0.062	0.02
A priori confounders	**Child sex**	**Male**	**[Reference]**	**–**	**–**	**[Reference]**	**–**	**–**
		**Female**	**0.47 (0.18, 0.76)**	**0.001**	**0.06**	**0.45 (0.13, 0.77)**	**0.006**	**0.05**
		Child age	0.02 (−0.13, 0.17)	0.818	0.00	0.00 (−0.16, 0.17)	0.973	0.00
		Maternal age	0.03 (−0.11, 0.18)	0.680	0.00	0.04 (−0.12, 0.20)	0.648	0.00

### Predictors of Adaptive Behavior

Least absolute shrinkage and selection operator regression models with adaptive behavior as the outcome consistently selected the child-rearing practice–nurturance subscale score and social support satisfaction across three LASSO models, with the maternal report of home stimulating level selected by only one model (Table 3). Nurturance and social support satisfaction scores were entered in a regression model with adaptive behavior as the outcome along with the *a priori* confounders (i.e., child age, child biological sex, and maternal age). Child-rearing nurturance showed a significant association with adaptive behavior, along with child and maternal age (Table 5). Specifically, when mothers scored an SD of 1 higher on the nurturance scale, children showed an SD of 0.25 (95% CL: 0.07–0.42) increase in adaptive behaviors. In contrast, child and maternal age were negatively associated with adaptive behavior scores as follows: child age β = −0.23 (95% CL: −0.39 to −0.07) and maternal age β = −0.21 (95% CL: −0.38 to −0.04). When adding the home stimulating level to the regression model, nurturance (β = 0.25, 95% CL: 0.07–0.44) and child age (β = −0.26, 95% CL: −0.41 to −0.10) remained significant at a similar magnitude of effect.

**Table 5:** Results from the adjusted model of LASSO-selected predictors for adaptive behavior with standardized scores.

			**Model 1 (** ***N*** **=** **137)**			**Model 2 (** ***N*** **=** **115)**		
			**R-Square: 0.178** ***p<0.0001***			**R-Square: 0.206** ***p*** **=** **0.0003**		
			**β** **(95% CI)**	***p***	**Partial** **η^2^**	**β** **(95% CI)**	***p***	**Partial** **η^2^**
LASSO selected		**CRPR nurturance**	**0.25 (0.07, 0.42)**	**0.007**	**0.05**	**0.25 (0.07, 0.44)**	**0.008**	**0.06**
		SSQ satisfaction	0.07 (−0.10, 0.24)	0.443	0.00	0.09 (−0.09, 0.27)	0.307	0.01
		Stimulating environment				0.10 (−0.07, 0.27)	0.254	0.01
A priori confounders	Child sex	Male	[Reference]	–	–	[Reference]	–	–
		Female	0.27 (−0.05, 0.59)	0.103	0.02	0.24 (−0.10, 0.58)	0.160	0.02
		Child age	–**0.23 (**–**0.39**, –**0.07)**	**0.004**	**0.06**	–**0.26 (**–**0.41**, –**0.10)**	**0.002**	**0.09**
		Maternal age	–**0.21 (0.38**, –**0.04)**	**0.015**	**0.04**	−0.11 (−0.30, 0.07)	0.220	0.01

## Discussion

This study explored the utility of measuring adaptive behavior, in addition to IQ, in studies of children at heightened risk for neurodevelopmental problems. Using the data from comprehensive assessments of preschoolers exposed to the Flint water crisis, we found that 71% of children demonstrated adaptive behavior skills at or above age level on the Vineland Adaptive Behavior Scales, while only 55% of children had measured IQ in the average or above range on the WPPSI-IV. These findings demonstrate the potential value of measuring adaptive behavior to capture additional capabilities not necessarily reflected by IQ scores. Furthermore, our analyses identified different socio-demographic correlates of adaptive behavior as compared with IQ, with implications for measuring developmental outcomes and targeting modifiable factors to improve these outcomes.

With regard to predictors of adaptive behavior, scores on the CRPR nurturance subscale, which is designed to capture positive parenting strategies and parent–child relationships (Rickel and Biasatti, 1982), emerged as a significant positive predictor with a medium effect size after accounting for *a priori* confounders. This is consistent with previous findings showing that parenting styles and behaviors impact the adaptive behavior of children (Altman and Mills, 1990; Rinaldi and Howe, 2012). Previous studies of young children suggest that caregivers with higher responsivity are more likely to develop positive relationships with their children and to facilitate gains in adaptive behaviors within a nurturing environment (Bradley et al., 1995; Glaser et al., 2003; Fenning and Baker, 2012; Warren et al., 2017). In contrast, other LASSO-selected predictors (i.e., social support satisfaction and stimulating home environment) did not show significant associations with adaptive behavior scores in the linear regressions. Taken together, these findings suggest that (1) positive mother–child relationships and interactions matter for the development of adaptive behaviors of children, and (2) mothers with less satisfying social support or limited resources and skills could still foster adequate adaptive skills in their children.

Different from the predictors of adaptive behavior, the predictors identified by the LASSO regression for IQ included maternal education and household income level, with maternal education level showing a significant association in the linear regression. This is consistent with previous studies showing that maternal education levels are a core factor in predicting child cognitive development (Harding et al., 2015; Jackson et al., 2017; Reardon, 2018). While income levels and maternal education are often correlated (in the current sample, mothers with more than high school education had higher household incomes, *t* = −2.79, *p* < 0.01), both are commonly found to be independently associated with child cognitive development (Tong et al., 2007; Hackman and Farah, 2009; Patra et al., 2016). The effect of SES on child intelligence has been attributed to better access to a stimulating and resourceful learning environment (e.g., books, toys, and learning activities) (Duncan and Brooks-Gunn, 2000; Tong et al., 2007; Christensen et al., 2014). It is likely mothers with higher educational levels are better equipped to provide an environment for promoting cognitive development (Dichtelmiller et al., 1992; Benasich and Brooks-Gunn, 1996; Winter et al., 2012).

The emergence of nurturance as a significant predictor of adaptive behavior suggests that interventions could potentially target nurturing practices among parents to improve the adaptive functioning of children (O'Connell et al., 2015; Roby et al., 2021). In fact, programs such as Reach Out and Read and the Video Interaction Project (Cates et al., 2016; Weisleder et al., 2019; Canfield et al., 2020) have been put in place to provide Flint parents with resources and training to promote positive parenting practices. Possible targets for improving the cognitive performance are less clear since SES factors are often a result of societal and structural challenges, and substantial long-term investment and intervention are often needed to bridge the cognitive performance gaps (Currie and Thomas, 1993; Campbell et al., 2002; Anderson et al., 2003; Love et al., 2005; Dobbie and Fryer, 2011).

We also observed associations between IQ and adaptive behavior and child sex and child age. Our findings on the advantage of females in IQ among Flint children are consistent with previous findings showing that males are at greater risk for neurodevelopmental disorders (Boyle et al., 2011) and more susceptible to environmental exposures (Jedrychowski et al., 2009; Chiu et al., 2017; DiPietro and Voegtline, 2017; Torres-Rojas and Jones, 2018). In addition, child age was negatively associated with adaptive behavior scores, suggesting that children may fall further behind the pace of the normative sample due to a cumulative effect of environmental disadvantages (Garcia Coll et al., 1998; Darbeda et al., 2018). The longitudinal data will be required to determine whether the relative strength in adaptive behavior for this sample persists as they grow into school age and beyond.

There are several limitations to be considered when interpreting the current findings. The sample size was relatively small, and all children were exposed to the Flint water crisis and multiple socioeconomic adversities. Therefore, replication in other samples is needed to determine the generalizability of these results. Our small sample size may also have resulted in limited power with which to detect statistically meaningful effects in the regression models. That is, candidate exposures that were not selected by the LASSO or were not significant in the linear regression should be interpreted with caution and not discounted in future research of IQ and adaptive behaviors. Moreover, given that the variances explained in the adaptive behavior and IQ models were small (*R*^2^: 0.12–0.21), more studies with longitudinal data and larger samples are needed to corroborate and extend our understanding of how adaptive behaviors and IQ change as a result of modifiable social environmental variables. Additionally, with the goal of reducing the burden of participants, we implemented a protocol change during the study resulting in the use of different versions of the Vineland scales with different data collection modalities (Vineland-II comprehensive interview vs. Vineland-3 parent-report survey). Although the ABC scores of high concurrent validity between Vineland-II and Vineland-3 has been reported (Sparrow et al., 2016), and we observed similar ABC score distributions (Supplementary Figure 1) and patterns (Supplementary Table 3) on the two versions in this study, the use of different forms to measure adaptive behavior is still a limitation. Another limitation is that our models included only binary maternal education levels. It is possible that more nuanced effects of maternal education levels could be detected if more levels of maternal education were considered in a larger sample. However, this study was the first to employ LASSO regression to select and then examine the correlates of adaptive behaviors in comparison to IQ. Our findings underscore the value of measuring adaptive behaviors in addition to IQ in studies of young children at heightened risk for neurodevelopmental difficulties.

## Data Availability Statement

The raw data supporting the conclusions of this article will be made available by the authors, without undue reservation.

## Ethics Statement

The studies involving human participants were reviewed and approved by Michigan State University, University of California, San Francisco. Written informed consent to participate in this study was provided by the participants' legal guardian/next of kin.

## Author Contributions

SZ, SB, and KL conceptualized the study. SZ conducted the statistical analysis. SZ and SB drafted the original manuscript. SB and KL provided major revisions to the drafts. TC, MH-A, and LO'C provided feedbacks and edits to the final manuscript. MH-A and KL secured the funding for the study. TC, SB, MH-A, and KL led the data collection. All authors contributed to the article and approved the submitted version.

## Conflict of Interest

The authors declare that the research was conducted in the absence of any commercial or financial relationships that could be construed as a potential conflict of interest.

## Publisher's Note

All claims expressed in this article are solely those of the authors and do not necessarily represent those of their affiliated organizations, or those of the publisher, the editors and the reviewers. Any product that may be evaluated in this article, or claim that may be made by its manufacturer, is not guaranteed or endorsed by the publisher.
