# Supplementary material for: Adaptive Behavior as an Alternative Outcome to Intelligence Quotient in Studies of Children at Risk: A Study of Preschool-Aged Children in Flint, MI, USA
Source: Front Psychol. 2021 Aug 11;12:692330. doi: 10.3389/fpsyg.2021.692330 (PMC8385490; doi:10.3389/fpsyg.2021.692330)
Supplement: Supplementary file 1 [file Table_1.docx]

**Supplemantary Table 1**

*Descriptive Statistics of Additional Predictors of Interests*

|  |  | **N** | **M (SD)** | **Range** |
| --- | --- | --- | --- | --- |
| CES-Depression | | 154 | 17.49(11.16) | [1,55] |
| PSS score | | 173 | 18.35(6.68) | [1,40] |
| CAGE-AID score | | 176 | 0.29(0.88) | [0,4] |
| LOT score | | 178 | 14.74(4.47) | [0,24] |
| SSQ Number score | | 179 | 2.25(1.66) | [0,9] |
| SSQ Satisfaction score | | 173 | 5.06(1.19) | [0.8,6] |
| HARK score | | 176 | 0.31(0.76) | [0,4] |
| CRPR Nurturance scale | | 156 | 71.78(14.9) | [1,90] |
| CRPR Conflict scale:  Often angry with my child | | 155 | 5.78(1.57) | [1,7] |
| CRPR Conflict scale:  Many conflicts between my child and me | | 153 | 5.9(1.75) | [1,7] |
| CRPR Conflict scale:  My child is a bit of a disappointment | | 154 | 6.47(1.38) | [1,7] |
| KEPS score | | 149 | 10.6(2.63) | [2,17] |
| NRI criticism score | | 155 | 1.19(0.33) | [0.33,2.33] |
| Stim-Q-P score | | 127 | 16.13(7.85) | [0,31] |
| ACE score | | 155 | 1.3(1.59) | [0,7] |
