# Supplementary material for: Adaptive Behavior as an Alternative Outcome to Intelligence Quotient in Studies of Children at Risk: A Study of Preschool-Aged Children in Flint, MI, USA
Source: Front Psychol. 2021 Aug 11;12:692330. doi: 10.3389/fpsyg.2021.692330 (PMC8385490; doi:10.3389/fpsyg.2021.692330)
Supplement: Supplementary file 2 [file Table_2.docx]

**Supplementary Table 2**

*Correlation Matrix of Exposure Variables on Parent-report Measures*

|  |  | 1 | 2 | 3 | 4 | 5 | 6 | 7 | 8 | 9 | 10 | 11 | 12 | 13 |
| --- | --- | --- | --- | --- | --- | --- | --- | --- | --- | --- | --- | --- | --- | --- |
| 1 | CES-Depression | 1 |  |  |  |  |  |  |  |  |  |  |  |  |
| 2 | PSS score | ***0.78*** | 1 |  |  |  |  |  |  |  |  |  |  |  |
| 3 | CAGE-AID score | 0.25 | 0.22 | 1 |  |  |  |  |  |  |  |  |  |  |
| 4 | LOT score | ***-0.46*** | ***-0.41*** | -0.12 | 1 |  |  |  |  |  |  |  |  |  |
| 5 | SSQ Number score | -0.14 | -0.16 | -0.11 | 0.12 | 1 |  |  |  |  |  |  |  |  |
| 6 | SSQ Satisfaction score | -0.24 | -0.22 | -0.13 | 0.16 | ***0.34*** | 1 |  |  |  |  |  |  |  |
| 7 | HARK score | 0.25 | 0.22 | 0.17 | 0.00 | -0.02 | -0.03 | 1 |  |  |  |  |  |  |
| 8 | CRPR Nurturance scale | -0.04 | -0.09 | -0.07 | 0.10 | 0.11 | 0.13 | -0.01 | 1 |  |  |  |  |  |
| 9 | CRPR Conflict scale | -0.12 | -0.02 | -0.12 | 0.09 | 0.04 | 0.05 | -0.07 | ***0.33*** | 1 |  |  |  |  |
| 10 | KEPS score | -0.03 | 0.02 | 0.07 | 0.08 | ***0.31*** | 0.08 | -0.03 | 0.25 | 0.10 | 1 |  |  |  |
| 11 | NRI criticism score | 0.02 | 0.08 | 0.21 | 0.06 | 0.03 | -0.01 | 0.08 | -0.15 | ***-0.33*** | -0.10 | 1 |  |  |
| 12 | Stim-Q-P score | -0.14 | -0.04 | -0.13 | 0.21 | -0.07 | 0.07 | -0.06 | 0.14 | 0.14 | 0.07 | -0.17 | 1 |  |
| 13 | ACE score | ***0.50*** | ***0.46*** | 0.32 | -0.21 | 0.01 | -0.07 | ***0.35*** | 0.04 | -0.01 | 0.19 | 0.08 | -0.10 | 1 |
| 14 | OECD modified income | -0.28 | -0.21 | -0.11 | 0.20 | 0.29 | 0.07 | -0.11 | 0.04 | 0.05 | ***0.30*** | 0.15 | -0.02 | -0.11 |
