# Supplementary material for: Adaptive Behavior as an Alternative Outcome to Intelligence Quotient in Studies of Children at Risk: A Study of Preschool-Aged Children in Flint, MI, USA
Source: Front Psychol. 2021 Aug 11;12:692330. doi: 10.3389/fpsyg.2021.692330 (PMC8385490; doi:10.3389/fpsyg.2021.692330)
Supplement: Supplementary file 3 [file Table_3.docx]

**Supplementary Table 3**

*Descriptive Statistics of Adaptive Behavior Composite Scores on Both Versions of Vineland Adaptive Behavior Scale in the Current Sample*

| **Vineland Version** | **N** | **Mean** | **Std Dev** | **Range** |
| --- | --- | --- | --- | --- |
| Vineland-2  Comprehensive Interview | 40 | 93.75 | 13.65 | [51,133] |
| Vineland-3 Parent Report Survey | 117 | 94.98 | 16.42 | [48,140] |

Note: Std Dev=Standard Deviation
