# Supplementary figures and images for: Adaptive Behavior as an Alternative Outcome to Intelligence Quotient in Studies of Children at Risk: A Study of Preschool-Aged Children in Flint, MI, USA
Source: Front Psychol. 2021 Aug 11;12:692330. doi: 10.3389/fpsyg.2021.692330 (PMC8385490; doi:10.3389/fpsyg.2021.692330)

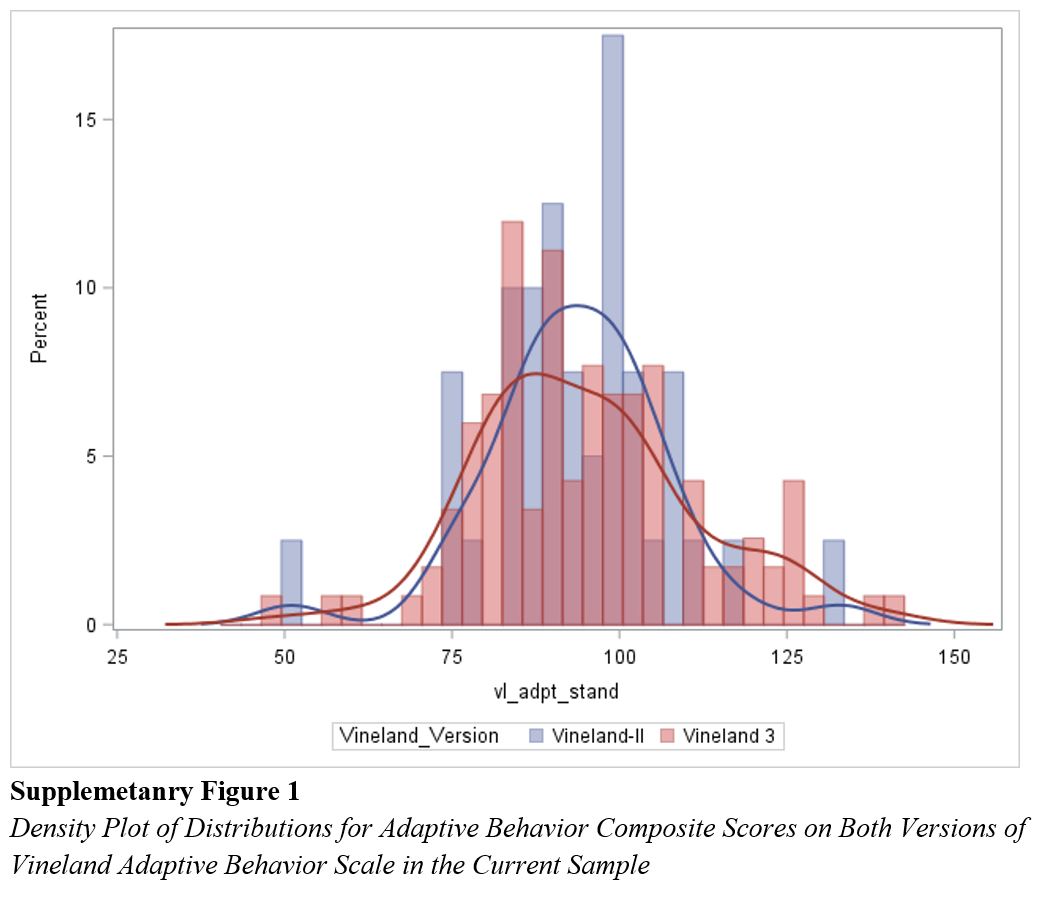

Supplement: Supplementary file 4 [file Image_1.jpg]
